# Breast Schwannoma

**DOI:** 10.1155/2011/930841

**Published:** 2011-02-09

**Authors:** Vandana Dialani, Neely Hines, Yihong Wang, Priscilla Slanetz

**Affiliations:** ^1^Department of Radiology, Beth Israel Deaconess Medical Center, 330 Brookline Avenue, Boston, MA 02215, USA; ^2^Radiology Associates of Hollywood, 9050 Pines Boulevard, Suite 200, Pembroke Pines, FL 33024, USA; ^3^Department of Pathology, Montefiore Medical Center and Albert Einstein College of Medicine, 1825 Eastchester Road, Bronx, NY 10461-2373, USA

## Abstract

Schwannomas arise from Schwann cells of the peripheral nerve sheath. The most common locations include the head, neck, and extensor surfaces of the extremities. Intramammary schwannomas are very rare and account for only 2.6% of schwannomas. A review of the English literature reveals 27 such cases of breast schwannoma. In this paper we describe another such rare case.

## 1. Case Report

**Figure 1:**
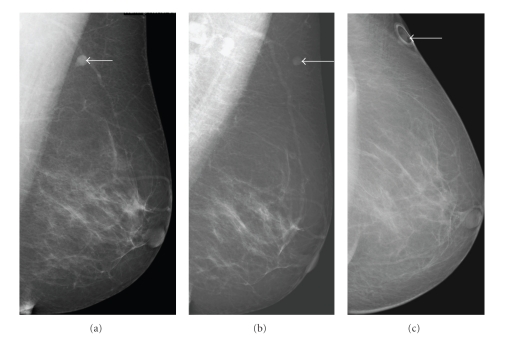
(a) Mediolateral view of the left breast shows an 8 mm well-defined ovoid mass in the left upper outer quadrant (arrow). The mass (arrow) has increased in size when compared to previous mammogram. (b) The mass (arrow) as seen on the previous years mammogram. (c) There is a skin marker overlying the mass (arrow) on the craniocaudal view.

**Figure 2:**
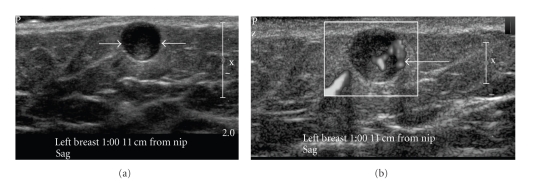
Ultrasound with a 17 MHZ linear probe. (a) demonstrates a 7 mm well-defined, complex hypoechoic mass within the breast superficially, abutting the skin (white arrows). (b) The mass shows significant central vascularity.

**Figure 3:**
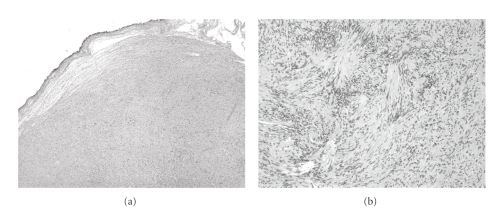
Histological examination. (a) shows an encapsulated mass consisting of monomorphic spindle cells with pointed basophilic nuclei (Antoni A tissue), set in a variable collagenous stroma (low power). Given limited excision of the encapsulated mass, adjacent normal breast parenchyma is not visualized. (b) shows areas of cells with parallel arrays of nuclear palisading known as Verocay bodies (high power).

A 64-year-old asymptomatic female presented for screening mammogram, which showed an 8 mm well-defined ovoid mass in the left upper outer quadrant. The benign appearing mass had increased in size compared to the screening mammogram one year prior (Figure 1). At the time of diagnostic evaluation, the radiologist noted a palpable nodule in the left upper outer quadrant, under the skin. Sonography demonstrated an 8 mm well-defined, vascular, complex hypoechoic mass within the breast superficially abutting the skin (Figure 2). Options of ultrasound-guided core needle biopsy and surgical excision were discussed, and the patient subsequently had a surgical excision. A well-encapsulated mass was removed from the breast. It measured 1.0 × 0.8 × 0.6 cm and was tan/white in color with focal cystic degeneration. Histological examination on low power revealed an encapsulated mass consisting of monomorphic spindle cells with pointed basophilic nuclei (Antoni A tissue), set in a variable collagenous stroma. Given limited excision of the encapsulated mass, adjacent normal breast parenchyma is not visualized (Figure 3(a)). On high power, areas of cells with parallel arrays of nuclear palisading known as Verocay bodies were noted (Figure 3(b)). Immunohistochemical stains were positive for S-100 protein, consistent with schwannoma.

Schwannomas arise from Schwann cells of the peripheral nerve sheath. The most common locations include the head, neck, and extensor surfaces of the extremities [1, 2]. Intramammary schwannomas accounted for only 2.6% of schwannomas in one series [3]. A review of the English literature shows, 27 such proven cases of breast schwannoma [1, 2, 4–20]. Most of them range from 7 mm to 11 cms. Our case is the second smallest case documented in the literature [12]. Mammographically, schwannomas are most commonly described as a nonspecific well-defined round or oval density [1, 2, 8]. A normal mammogram and an ill-defined mass have also been reported [12]. Sonographically, more variation in appearance has been reported; however it is most commonly reported as a solid hypoechoic well-defined mass with variable posterior acoustic enhancement [1, 8], as seen in our case.

Microscopically, classic schwannoma is an encapsulated neoplasm having two components known as Antoni A tissue and B tissue, in variable proportions. Antoni A tissue is cellular and consists of monomorphic spindle-shaped Schwann cells, with poorly defined eosinophilic cytoplasm and pointed basophilic nuclei, set in a variable collagenous stroma [1]. These cells commonly show nuclear palisading and parallel arrays of such palisades with intervening eosinophilic cell cytoplasm (processes) are known as Verocay bodies [21].

Breast schwannomas show no definite worrisome mammographic or ultrasonographic features, and an imaging diagnosis is impossible. A diagnosis of schwannoma of the breast may be suggested on a core needle biopsy if there is a cytologically bland spindle cell lesion with areas of palisading, lack of epithelial elements, especially if the cells show immunostaining for S-100 protein [21]. However, distinction from other spindle cell lesions such as metaplastic carcinomas, a fibroepithelial lesion with minor epithelial components, fibromatosis, myofibroblastoma, among others, will likely require an excisional biopsy [12, 21].
